# Social Adversity and Triple-Negative Breast Cancer Incidence Among US Black Women

**DOI:** 10.1001/jamanetworkopen.2025.37378

**Published:** 2025-10-14

**Authors:** Alexandra E. Hernandez, Angela Mazul, Neha Goel

**Affiliations:** 1 Division of Surgical Oncology, Department of Surgery, University of Miami Miller School of Medicine, Miami, Florida; 2 Sylvester Comprehensive Cancer Center, University of Miami Miller School of Medicine, Miami, Florida; 3 UPMC Hillman Cancer Center, Pittsburgh, Pennsylvania; 4 Department of Otolaryngology, University of Pittsburgh Medical Center, Pittsburgh, Pennsylvania; 5 Breast Service, Department of Surgery, Memorial Sloan Kettering Cancer Center, New York, New York

## Abstract

This cohort study investigates the association between social adversity and incidence of triple-negative breast cancer among US Black women.

## Introduction

Triple-negative breast cancer (TNBC) is an aggressive subtype associated with shorter survival that disproportionately affects non-Hispanic Black women.^1,2^ Social adversity, including exposure to chronic social, economic, and environmental stressors, is associated with higher odds of TNBC independent of established individual-level risk factors.^1^ Regional studies identified that Black women with lower vs higher social adversity have increased odds of TNBC.^1,2,3,4^ This study aimed to validate the association between social adversity and TNBC incidence in a US cohort of Black women.

## Methods

This cohort study included Surveillance, Epidemiology, and End Results (SEER) 18 (2006-2020) data based on the Yost Index, a census tract–level measure of socioeconomic status developed for cancer surveillance. As data are deidentified and publicly available, local ethics review and informed consent were not required in accordance with the Common Rule. The study followed the STROBE reporting guideline.

Black women diagnosed with stage I to IV TNBC between 2010 and 2020 (*ERBB2* [formerly *HER2* or *HER2*/neu] status was not available before 2010) were included. The primary outcome was age-adjusted incidence of TNBC per 100 000 persons. Incidence rates age adjusted to the 2000 US standard population were calculated using SEER*Stat, version 8.3.9 (National Cancer Institute). We also calculated incidence rate ratios with 95% CIs, comparing each Yost quintile with the most disadvantaged group. Significance was considered when the 95% CI did not include 1. Incidence of TNBC by county was mapped using SEER 22. Data were analyzed between January 8, 2024, and August 8, 2025.

## Results

Overall, 13 340 Black women were included (mean [SD] age, 58.1 [12.8] years). The age-standardized TNBC incidence rate was highest in the highest social adversity category (group 1) (19.3 [95% CI, 18.8-19.9] per 100 000 persons) and incrementally decreased by each Yost Index group (Table). The Figure shows counties with the highest poverty levels and incidence of TNBC among Black women.

**Table.** Incidence Rates for TNBC in Non-Hispanic Black Women by Yost Index Quintile

Yost index quintile	Incidence rate (95% CI)^a^	Count, No.	IRR (95% CI)	Population, No.
Group 1 (lowest nSES)	19.3 (18.8-19.9)	4930	1 [Reference]	25 664 865
Group 2	17.9 (17.2-18.6)	2758	0.93 (0.88-0.97)	15 667 272
Group 3	18.2 (17.4-19.0)	2189	0.94 (0.90-0.99)	12 295 342
Group 4	19.0 (18.2-19.9)	2123	0.98 (0.93-1.04)	11 088 640
Group 5 (highest nSES)	17.2 (16.3-18.2)	1340	0.89 (0.84-0.95)	7 559 541

Abbreviations: IRR, incidence rate ratio; nSES, neighborhood socioeconomic status; TNBC, triple-negative breast cancer.

^a^ Rates are per 100 000 persons and age adjusted to the 2000 US standard population. The 95% CIs for rates are by Tiwari modification.

**Figure.**
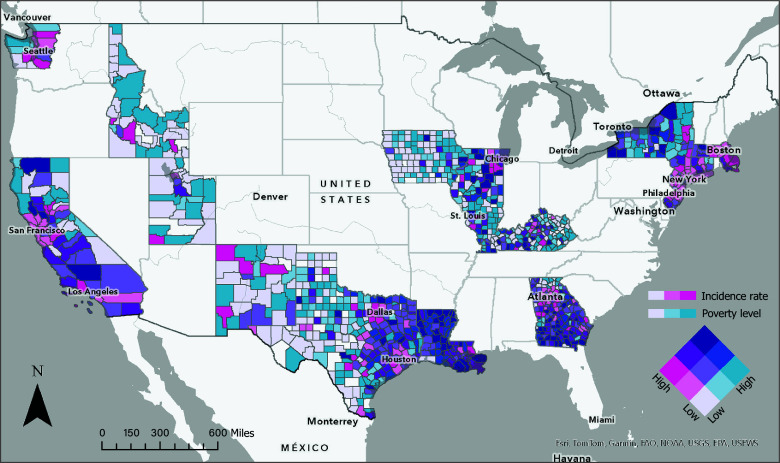
Age-Adjusted Incidence of Triple-Negative Breast Cancer and Socioeconomic Disadvantage in Non-Hispanic Black Women in a National Cohort, 2010 to 2020 County-level shading reflects the overlap between age-adjusted poverty rates and triple-negative breast cancer incidence. Map created with ArcGIS Pro, version 3.2 (Esri).

## Discussion

This cohort study shows that high social adversity is associated with a high incidence of TNBC among Black women. As subtype is intrinsic to tumor development, these findings suggest an early and critical source of downstream breast cancer survival disparities. Our findings suggest potential social (epi)genomic and gene-environment interactions between social adversity and TNBC development in Black women.^4,5^

Disadvantaged neighborhoods are a byproduct of social, nutritional, and environmental disinvestment secondary to political policies, eg, historical redlining. Low neighborhood socioeconomic status is associated with chronic stress. Social genomic studies have uncovered associations between social adversity stressors, eg, threats to safety and subsequent sympathetic nervous system activation, and more aggressive breast cancer tumor biology.^4^ Social epigenomic studies identified associations between social adversity and differential DNA methylation (DNAm) of proinflammatory genes and epigenetic acceleration. Social adversity may affect the epigenome through nutritional intake of cofactors involved in DNAm and chromatin remodeling (folate, B12, vitamin C).^5^ Women living in social adversity often are exposed to superfund sites and elevated toxin concentrations, including endocrine-disrupting toxins (bisphenol A, dioxins) that may influence global DNAm and promote proliferation of cancer cells, along with potential gene-environment interactions.^5^

Reversing these structural inequities will require targeted investment in historically disadvantaged neighborhoods, including enforcing environmental regulations, expanding access to nutritious foods, and funding community safety initiatives. Policies that address redlining through equitable housing and infrastructure investment may help dismantle the social and environmental conditions that contribute to TNBC disparities.

This study is limited by the absence of individual-level data (eg, *BRCA1* variant status, family history, reproductive factors), which may have introduced residual confounding and potential for ecologic fallacy. Furthermore, the Yost Index does not fully encapsulate neighborhood-level social adversity.

Taking a translational epidemiologic approach to identify modifiable risk factors of TNBC is important.^5^ Geospatial visualization could inform future studies evaluating county-level gene-environment or social (epi)genomic risk factors (proximity to superfund sites) and which high-risk counties should implement cancer control interventions, such as heightened surveillance, to identify TNBC incidence.^6^ These strategies may overcome disparate breast cancer survival outcomes among US Black women.

## Notes

Supplement.
Data Sharing Statement
