# Supplementary material for: Social Adversity and Triple-Negative Breast Cancer Incidence Among US Black Women
Source: JAMA Netw Open. 2025 Oct 14;8(10):e2537378. doi: 10.1001/jamanetworkopen.2025.37378 (PMC12522001; doi:10.1001/jamanetworkopen.2025.37378)
Supplement: Supplement. — Data Sharing Statement [file jamanetwopen-e2537378-s001.pdf]

Data Sharing Statement

Hernandez. Social Adversity and Triple-Negative Breast Cancer Incidence Among US Black Women. *JAMA Netw Open*. Published October 14, 2025.
doi:10.1001/jamanetworkopen.2025.37378

Data
Data available: No

Additional Information
Explanation for why data not available: National data others can already obtain
